# Faculty of Medicine in Geneva

**Published:** 1874-06

**Authors:** 


The New Faculty of Medicine in Geneva.—The Grand Council of Geneva has sanctioned the creation of this new faculty in the Academy of Geneva, which is henceforth to be known as the University of Geneva. The curriculum will embrace the entire circle of theoretical and clinical medicine and surgery.—*New York Medical Journal.*

